# Gemcitabine-Containing Chemotherapy for the Treatment of Metastatic Myxofibrosarcoma Refractory to Doxorubicin: A Case Series

**DOI:** 10.3390/curroncol28010078

**Published:** 2021-02-05

**Authors:** Arielle Elkrief, Suzanne Kazandjian, Thierry Alcindor

**Affiliations:** 1 Cedars Cancer Center, McGill University Health Centre, Montreal, QC H4A 3J1, Canada; arielle.elkrief@mail.mcgill.ca; 2 Department of Medicine, McGill University Health Centre, Montreal, QC H4A 3J1, Canada; suzanne.kazandjian@mail.mcgill.ca

**Keywords:** myxofibrosarcoma, sarcoma, chemotherapy

## Abstract

Background: Myxofibrosarcoma is a type of soft-tissue sarcoma that is associated with high rates of local recurrence and distant metastases. The first-line treatment for metastatic soft-tissue sarcoma has conventionally been doxorubicin-based. Recent evidence suggests that myxofibrosarcoma may be molecularly similar to undifferentiated pleomorphic sarcoma (UPS), which is particularly sensitive to gemcitabine-based therapy. The goal of this study was to evaluate the activity of gemcitabine-containing regimens for the treatment of metastatic myxofibrosarcoma refractory to doxorubicin. Material and Methods: We retrospectively evaluated seven consecutive cases of metastatic myxofibrosarcoma at our institution treated with gemcitabine-based therapy in the second-line setting, after progression on doxorubicin. Baseline clinical and baseline characteristics were collected. Primary endpoints were objective response rate (ORR), progression-free survival (PFS) and overall survival (OS). Results: After progression on first-line doxorubicin, a partial, or complete radiological response was observed in four of seven patients who received gemcitabine-based chemotherapy. With a median follow-up of 14 months, median progression-free and overall survival were 8.5 months and 11.4 months, respectively. Conclusions: Gemcitabine-based chemotherapy was associated with encouraging response rates in this cohort, similar to those seen in UPS. Both entities could be studied together for novel gemcitabine-based regimens.

## 1. Introduction

Soft-tissue sarcomas are a relatively rare tumor group and first-line treatment for metastatic disease has conventionally been doxorubicin-based [1]. Although there have been notable advances in systemic therapies in the past years, prognosis still remains poor with advanced disease, with studies reporting a median overall survival (OS) between 14 and 17 months [2]. 

Given the rarity of soft-tissue sarcoma, subgroups have usually been studied altogether for potential therapies. However, recent evidence has shown that systemic therapy response differs depending on histology. Notably, undifferentiated pleomorphic sarcomas (UPS) and leiomyosarcomas are sensitive to regimens containing gemcitabine [3].

Myxofibrosarcoma (MFS) is another subtype of soft-tissue sarcoma which usually arises in the extremities and is associated with high rates of local recurrence and distant metastases [4]. Tumor grade, percentage of myxoid component, and positive margins after resection are predictors of recurrence. Median progression-free survival (PFS) of advanced myxofibrosarcoma treated with anthracycline-based therapy is four months, and response rates to second-line chemotherapy are reported to be as low as 10% [5]. Because of sparse data, there is currently no consensus on second-line treatment in the metastatic setting. Recent studies suggest that MFS may be molecularly similar to UPS [6], which is particularly sensitive to gemcitabine-based therapy [6]. However, the activity of gemcitabine-based therapy in MFS remains unknown. Therefore, the goal of this study was to evaluate the role of gemcitabine-containing regimens for the treatment of metastatic myxofibrosarcoma refractory to doxorubicin.

## 2. Material and Methods

Between 2010 and 2019, a total of 17 cases of metastatic MFS refractory to doxorubicin were identified and retrospectively evaluated at the McGill University Health Centre (MUHC). Inclusion criteria were a diagnosis of metastatic MFS on second- line treatment with gemcitabine-based therapy after progression on doxorubicin; availability of radiological and clinical data for assessment of response. Ten patients were excluded due to lack of follow-up, other agent used as second-line therapy or no further treatment offered after doxorubicin. A total of 7 patients were included in our cohort (Figure S1 for Consort diagram). Clinical data were retrieved retrospectively from the MUHC sarcoma database and included age, sex, performance status, location and size of initial tumor, stage, status of margin after resection, histological grade, depth of tumor, area of metastasis, treatment agent, time of recurrence, death, last follow-up, and response rate at last follow-up. Primary outcomes were objective response rate (ORR), PFS, and OS. PFS was defined as the time of first administration of gemcitabine-based therapy until objective radiological disease progression according to the RECIST criteria or death, and overall survival was defined as the time until last follow-up or death. The study was approved by the MUHC REB Ethics Committee (Ethics number 2021-7060).

## 3. Results

The baseline characteristics of all seven patients are summarized in Table 1. Median age was 66 years, and 43% of patients were male. Six patients had a performance status of less than one. All patients had a diagnosis of myxofibrosarcoma originating in the lower extremities with previous primary surgical resection. As per the American Joint Committee on Cancer R classification criteria, five of seven had margins which were involved after primary resection (R1) and four of them had deep margins involved. More than half of the patients had high-grade disease on histology. 

**Figure 1:**
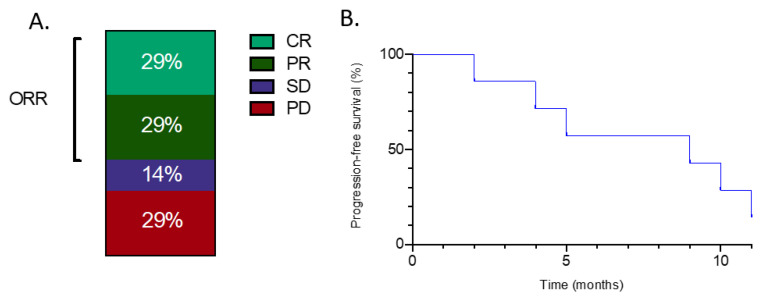
(**A**) Objective response rates with gemcitabine-containing regimens for patients with metastatic myxofibrosarcoma treated in the second-line setting. ORR: Objective response rate. PD; progressive disease. CR; complete response SD; Stable disease PR; Partial response. (**B**) Progression-free survival with gemcitabine-containing regimens for patients with metastatic myxofibrosarcoma treated in the second-line setting.

One of seven patients received gemcitabine with docetaxel as a second-line treatment, five patients received gemcitabine and dacarbazine and one received gemcitabine as a single agent. Four of seven patients showed either a partial or a complete radiological response (Figure 1A.) One patient (case 3) with oligometastatic lung involvement experienced partial response (PR) and underwent consolidative stereotactic body radiotherapy to the remaining lung nodule, resulting in a prolonged disease-free status. Another one (case 7) with complete resolution of lung nodules for 10 months had an aggressive disease recurrence and died one month later. With a median follow-up of 14 months, median progression-free and overall survival were 8.5 months and 11.4 months, respectively (Figure 1B.)

The American Joint Committee on Cancer R classification R0: wide negative margin. R1: microscopically positive margin. R2: macroscopically positive margin.

## 4. Discussion and Conclusions

MFS and UPS, both formerly known as malignant fibrous histiocytoma, can be difficult to distinguish. Some authors have proposed that a threshold of at least 10% of myxoid component be an attribute of MFS [7]. It is thought that the hypocellular myxoid component predisposes patients to local recurrence. With recurrence, MFS can become more cellular and resemble UPS morphologically, with no difference in overall survival between recurrent MFS with myxoid area of <10% and primary UPS [7]. Indeed, The Cancer Genome Atlas (TCGA) sarcoma genetically analyzed different subtypes of sarcomas and concluded that, on a molecular basis, UPS and MFS are a spectrum of the same pathology [8]. Although the biology of MFS and UPS is still poorly understood [9], next-generation sequencing in a study of 94 pathology samples of patients diagnosed with either disease revealed frequent loss of *Rb* and *p53*, two tumor suppressor genes, from chromosomal deletions or loss-of-function mutations, in both entities [10]. MFS and UPS presenting with these mutations rely on *Skp2*, an oncogene that promotes cell turnover, to proliferate, and thus Li et al. suggest targeting *Skp2* in order to suppress the cancer with novel therapies. Gemcitabine, an antimetabolite that incorporates into DNA and blocks replication, has shown its activity in tumor cells presenting with loss of function for *p53* in different tumor subgroups [11]. 

A phase II randomized trial comparing gemcitabine monotherapy to gemcitabine with docetaxel in metastatic soft-tissue sarcomas refractory to initial treatment showed superiority in terms of overall survival and progression-free survival with the combination [3]. The response to combination therapy was particularly notable in the leiomyosarcoma and UPS subgroups, and therefore this is generally the preferred second-line regimen in recurrent UPS. Similarly, a phase II randomized study comparing gemcitabine with dacarbazine versus dacarbazine alone in patients with recurrent soft-tissue sarcoma revealed superior median overall survival of 16.8 months compared to 8.2 months in the combined regimen [12]. Gemcitabine has thus a role in previously treated soft-tissue sarcomas, especially UPS and leiomyosarcoma, but to our knowledge, there are no current data evaluating the response of myxofibrosarcoma specifically to gemcitabine. As such, our study showed that gemcitabine in second-line setting in metastatic myxofibrosarcoma was associated with encouraging response rates, similar to those observed in UPS, further hinting at the similarities between these two sarcoma types. The exact contribution of docetaxel or dacarbazine to the activity of gemcitabine-based combinations as second-line treatment is unclear, and it is not known whether one is superior to the other in that setting. In our institution, we have noted greater convenience and better tolerability with the gemcitabine/dacarbazine regimen, hence our frequent use of it. 

Regarding newer treatments, immunotherapy with pembrolizumab is active in UPS, as shown in a prospective multicenter study [13]. Although there are no comparable data for MFS, some case reports are also suggestive of the potential benefit of immune checkpoint inhibitors in this disease [14,15]. Furthermore, the Melanoma-associated antigen 3 (MAGE-A3) is expressed in both UPS and MFS [16] and is, therefore, a potential immunotherapy target for both entities.

On the basis of the above-mentioned relationship between UPS and MFS and the promising response of MFS to gemcitabine, we agree that both entities could be studied together for novel gemcitabine-based regimens, as already done by some investigators [17].

## Figures and Tables

**Table 1:** Baseline characteristics.

Variation	Age	Sex	ECOG	Size (cm)	Depth	Grade	Margins	Regimen	Best Response	PFS	OS
Case 1	66	M	2	Unknown	Unknown	2	Unknown	Gemcitabine/docetaxel	PD	1.5 mo	2 mo
Case 2	68	M	1	16.5	Deep	3	Affected-R1	Gemcitabine/dacarbazine	PD	5 mo	11 mo
Case 3	68	M	0	17	Deep	2	Affected-R1	Gemcitabine	PR	8.5 mo	83 mo
Case 4	62	F	1	15	Deep	3	Affected-R1	Gemcitabine/dacarbazine	SD	12 mo	13 mo
Case 5	67	F	1	2.6	Superficial	3	Affected-R1	Gemcitabine/dacarbazine	PR	11 mo	13 mo
Case 6	66	F	1	14.5	Deep	3	Affected-R1	Gemcitabine/dacarbazine	PR	4 mo	8 mo
Case 7	63	F	1	9	Deep	3	Uninvolved-R0	Gemcitabine/dacarbazine	CR	10 mo	11 mo

PD: progressive disease. CR: complete response. SD: Stable disease. PR: Partial response.

## Supplementary Materials

The following are available online at https://www.mdpi.com/1718-7729/28/1/78/s1, Figure S1: Consort diagram for study inclusion.
