# Supplementary material for: Gemcitabine-Containing Chemotherapy for the Treatment of Metastatic Myxofibrosarcoma Refractory to Doxorubicin: A Case Series
Source: Curr Oncol. 2021 Feb 5;28(1):813–7. doi: 10.3390/curroncol28010078 (PMC7985763; doi:10.3390/curroncol28010078)
Supplement: Supplementary file 1 [file curroncol-28-00078-s001.pdf]

Current Oncology

Brief Report

Gemcitabine-Containing Chemotherapy for the Treatment of Metastatic Myxofibrosarcoma Refractory to Doxorubicin: A Case Series

Arielle Elkrief, Suzanne Kazandjian and Thierry Alcindor

Supplementary Materials:

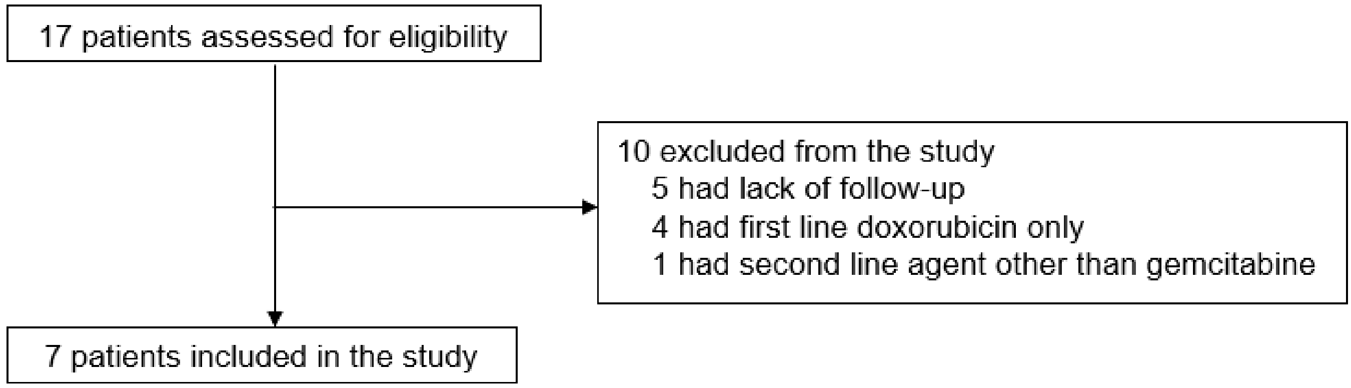

Figure S1. Consort diagram for study inclusion.
